# Expert Consensus on Effective Management of Chemotherapy-Induced Nausea and Vomiting: An Indian Perspective

**DOI:** 10.3389/fonc.2020.00400

**Published:** 2020-03-27

**Authors:** Ashok K. Vaid, Sudeep Gupta, Dinesh C. Doval, Shyam Agarwal, Shona Nag, Poonam Patil, Chanchal Goswami, Vikas Ostwal, Sagar Bhagat, Saiprasad Patil, Hanmant Barkate

**Affiliations:** ^1^Medical Oncology and Hematology, Medanta – The Medicity, Gurugram, India; ^2^ACTREC-TATA Memorial Centre, Navi Mumbai, India; ^3^Medical Oncology, Rajiv Gandhi Cancer Institute and Research Centre, New Delhi, India; ^4^Medical Oncology, Sir Ganga Ram Hospital, New Delhi, India; ^5^Medical Oncology, Sahyadri Hospital, Pune, India; ^6^Medical Oncologist, Manipal Hospital, Bangalore, India; ^7^Oncology Services, MEDICA Super Speciality Hospital, Kolkata, India; ^8^Medical Oncology, TATA Memorial Hospital, Mumbai, India; ^9^Medical Services, HO IF, Glenmark Pharmaceuticals Ltd., Mumbai, India; ^10^Medical Services, IF, Glenmark Pharmaceuticals Ltd., Mumbai, India; ^11^Medical Services, IF & MEA, Glenmark Pharmaceuticals Ltd., Mumbai, India

**Keywords:** CINV, risk scoring, antiemetics, consensus, quality of life

## Abstract

Chemotherapy-induced nausea and vomiting (CINV) is one of the most common and feared side effects in cancer patients undergoing chemotherapy. Scientific evidence proves its detrimental impact on a patient's quality of life (QoL), treatment compliance, and overall healthcare cost. Despite the CINV-management landscape witnessing a radical shift with the introduction of novel, receptor-targeting antiemetic agents, this side effect remains a chink in the armor of a treating oncologist. Though global guidelines acknowledge patient-specific risk factors and chemotherapeutic agent emetogenic potential in CINV control, a “one-fit-for-all” approach cannot be followed across all geographies. Hence, in a pioneering attempt, India-based oncologists conveyed easily implementable, region-specific, consensus-based statements on CINV prevention and management. These statements resulted from integrating the analysis of scientific evidence and guidelines on CINV by the experts, with their clinical experience. The statements will strengthen decision-making abilities of Indian oncologists/clinicians and help in achieving consistency in CINV prevention and management in the country. Furthermore, this document shall lay the foundation for developing robust Indian guidelines for CINV prevention and control.

## Introduction

Chemotherapeutic approach for cancer is associated with the management of various adverse effects, which poses a great challenge to healthcare providers, thus having a detrimental impact on a patient's overall QoL (1, 2). Scientific evidence over time reveals nausea and vomiting to be the two most frequent and feared, yet underestimated, side effects in patients receiving chemotherapy (3–7). Physiologically, uncontrolled/poorly controlled and prolonged CINV leads to malnutrition, dehydration, and electrolyte imbalance. These adverse effects further lead to complications such as esophageal tears and declining behavior (toward the treatment) (8). The physiological distress caused by CINV further transcends by negatively affecting a patient's ability to carry out normal daily activities/chores (9).

Severe and poorly controlled CINV was ranked near death by patients undergoing chemotherapy (6). Chemotherapy-induced nausea and vomiting not only has the propensity to increase morbidity, and healthcare cost, but also interferes with the chemotherapy adherence and patient's QoL (9–17). The current antiemetic agents exert their action by targeting various receptors [5-hydroxytryptamine (5-HT3), neurokinin 1 (NK1), dopamine, etc.] involved in the emesis mechanism (18).

Even though current global guidelines acknowledge the emetogenic potential of chemotherapeutic agents and patient-specific risk factors, management of delayed emesis is still a battle un-won. The Indian oncologists largely depend upon the National Comprehensive Cancer Network (NCCN) recommendations, the American Society of Clinical Oncology (ASCO) clinical updates and European society for medical oncology/multinational Association of Supportive Care in Cancer (ESMO/MASCC) recommendations. However, it is pertinent to state that these global guidelines are tailored according to the functioning of the healthcare setups in developed nations and do not account for factors unique to the healthcare systems of developing countries (19). Healthcare dynamics in a developing economy as ours are different from those of the developed nations. Healthcare accessibility, coupled with issues such as variable management practices and lack of sensitization for guidelines, has made the development of region-specific CINV-management guidelines the need of the hour (19). Findings from previously conducted multination Pan Australasian ChemoTherapy InduCed Emesis burden of illness (PrACTICE) study reported vast variation in the complete response (CR) rate (~50–87%) among patients from different participating countries (20).

India reported a better overall CR rate when compared to Australia, China, and Singapore. On the other hand, Australia reported higher proportions of patients with no emesis compared to other Asian countries. Additionally, Asian countries, including India, reported high use and prescribing behavior of CINV rescue medication (20). Considering the response variations of various antiemetic agents, region-specific management guidelines are the need of the hour. Structuring region-specific recommendations for CINV will acknowledge the patient-related risk factors, affordability, sociocultural influence, and prevalent clinical oncology practice aspects in the country.

Hence, in a pioneering attempt, this document aims at guiding Indian oncologists on effective management of CINV in clinical practice.

## Methodology

### Consensus Development Process

The consensus-based clinical statements (Table 1) presented in the document were developed by the cumulative efforts of 45 oncologists, of whom eight oncologists constituted the core expert group. The initial inputs were gathered from the core group committee face-to–face interaction in August 2018. The clinical statements were validated, and then responses were gathered from the core expert group. Modified Delphi methodology was applied to achieve consensus on the initial votes from the core group. Following the initial votes, inputs from 35 India-based oncologists were taken through a Google survey link, using a 5-point Likert scale, to measure the cumulative agreement on 45 clinical statements. These inputs were received in September 2018. The anonymity of the participating oncologists was duly maintained. The 5-point Likert scale reads as follows:

**Table 1:** Summary of clinical consensus statements.

**S. No**	**Clinical statements**	**Mean score**	**Level of evidence and grade of recommendation**
1	The risk for CINV depends not only on the type of chemotherapy administered but also on the patient's profile.	4.2	1A
2	Risk of chemotherapy induced nausea vomiting is higher during the first two cycles of chemotherapy.	3.8	2A
3	Nausea and vomiting in the previous cycle are a significant predictor of subsequent and clinically significant nausea and/or vomiting.	4.2	1A
4	Anxiety increases the risk of nausea and vomiting in patients scheduled for chemotherapy.	4.4	1A
5	History of motion sickness is important predictors of CINV.	3.8	2A
6	History of morning sickness in pregnancy increases patients' risk of CINV.	3.5	2A
7	Concomitant radiotherapy increases the risk of CINV in patients undergoing chemotherapy.	4.0	1C
8	Patients with poor performance status especially due to the disease process (ECOG status > 1) are more likely to experience nausea and vomiting.	3.9	2C
9	Females are at a higher risk of nausea vomiting (both acute and delayed) than males.	4.1	1A
10	Younger patients, <60 years of age, the risk of nausea vomiting is high.	3.4	2A
11	Pain and Cancer-related fatigue increases patients' risk of nausea and vomiting.	3.8	2C
12	Lack of sleep, the night previous to chemotherapy, increases the risk of nausea vomiting.	4.0	1C
13	Low or no alcohol intake is an independent risk factor for nausea vomiting. (both acute and delayed).	3.5	2A
14	Non-smokers are at a higher risk for nausea vomiting. (both acute and delayed).	2.9	2C
15	Risk of nausea- vomiting increases in patients on alternative (homeopathic/ayurvedic) medications.	3.0	2C
16	The risk of CINV increases if the patient is bombarded with the thought of CINV by family members.	4.0	1C
17	Classification of intravenous chemotherapeutic agents by NCCN guideline 2018 into HEC /MEC/LEC/Minimal is comprehensive.	4.1	1A
18	Cisplatin irrespective of the dose and regimen should be considered as HEC.	3.7	2A
19	AC combination should be considered as HEC.	4.0	1A
20	Carboplatin combination should be considered as HEC.	3.5	2B
21	Oxaliplatin combination should be considered as HEC.	3.0	2C
22	NK1RA needs to be used as a dexa sparing regime, especially while administering oxaliplatin in dextrose in patients with uncontrolled or poorly controlled diabetes.	3.4	2C
23	Netupitant being CYP3A4 inhibitor, expected to increase the exposure (AUC) of oral dexamethasone; hence, reduction in oral dexamethasone dose can be adapted during co-administration (from 20 to 12 mg).	3.9	2A
24	ECG monitoring is essential in patients on 5HT3 RA, considering the increased risk of QT prolongation associated with 5HT3 RA.	3.5	2A
25	Fear of QTc prolongation with antiemetics regimen is spurious.	3.6	2C
26	Patients who receive HEC—for controlling acute CINV (day 1), should be treated with triple combination therapy containing 5HT3 RA, dexamethasone and NK1 RA.	4.3	1A
27	Patients who receive HEC—for controlling acute CINV (day 1), should be treated with four drug combination therapy containing 5HT3 RA, dexamethasone, NK1 RA and olanzapine.	3.5	2A
28	Patients who receive HEC—for controlling delayed CINV (days 2–5), should be treated with dual therapy containing NK1 RA and dexamethasone.	3.7	2A
29	Patients who receive HEC—for controlling delayed CINV (days 2–5), should be treated with dual therapy containing olanzapine and dexamethasone.	3.3	2A
30	Patients who receive HEC—palonosetron is the preferred 5-HT3 antagonist.	3.8	2A
31	Patients who receive HEC—increased drowsiness is a worrisome side effect with olanzapine.	3.6	2C
32	Patients who receive MEC—for controlling acute CINV (day 1), should be treated with dual therapy containing 5HT3 RA and dexamethasone.	4.0	1A
33	Patients who receive MEC—for controlling delayed CINV, triple therapy with NK1RA improves outcome.	4.1	1A
34	Patients who receive MEC—for controlling delayed CINV (days 2–5), should be treated with dexamethasone only.	3.0	2A
35	Patients who receive MEC—for controlling delayed CINV (days 2–5), should be treated with NK1RA + dexamethasone.	3.3	2B
36	Patients who receive MEC—for controlling delayed CINV (days 2–5), patients should be treated with olanzapine + dexamethasone.	3.3	2C
37	Patients who receive LEC and minimally emetogenic regimen—for controlling acute and delayed CINV, patients should be treated with dexamethasone only on day 1.	3.4	2A
38	Patients who receive minimally emetogenic regimen—needs no treatment to prevent CINV.	3.2	2A
39	Patients who receive multiday chemotherapy—long acting NK1RA to be given only on day 1.	3.8	2A
40	Patients who receive multiday chemotherapy—long acting NK1 RA should be given on days 1,3, and 5.	2.8	2C
41	Patients who receive multiday chemotherapy−5-HT3 receptor antagonist should be given daily.	3.5	2A
42	Patients who receive multiday chemotherapy–palonosetron, should be given on days 1, 3, and 5.	3.1	2A
43	Benzodiazepines are the only agents that have been shown to reduce the incidence of anticipatory nausea and vomiting.	3.7	2A
44	Olanzapine is the drug of choice in patients with through CINV.	3.4	2A
45	Sedation associated with olanzapine can be useful in the overall management of CINV.	3.4	2B

*AC, Adriamycin-cyclophosphamide; AUC, Area under the curve; CINV, Chemotherapy-induced nausea and vomiting; ECOG, Eastern Cooperative Oncology Group; HEC, Highly emetogenic chemotherapy; MEC, Moderately emetogenic chemotherapy; LEC, Low emetogenic chemotherapy; NCCN, National Comprehensive Cancer Network; NK1 RA, Neurokinin-1 receptor antagonist; 5-HT3 RA, 5-hydroxytryptamine receptor antagonists*.

**Strongly disagree: Score of 1; Disagree: Score of 2; Neutral: Score of 3, Agree: Score of 4; Strongly agree: Score of 5**.

The consensus-based statements were categorized as follows:

- Consensus: A mean score of ≥4 was considered as a consensus agreement.
- Near Consensus: A mean score of 3 to <4 was considered a near consensus agreement. Institutional and regional clinical practice may be considered for such statements.
- No consensus: Statements that did not meet the criteria of consensus or near consensus statements.

**Figure 1:**
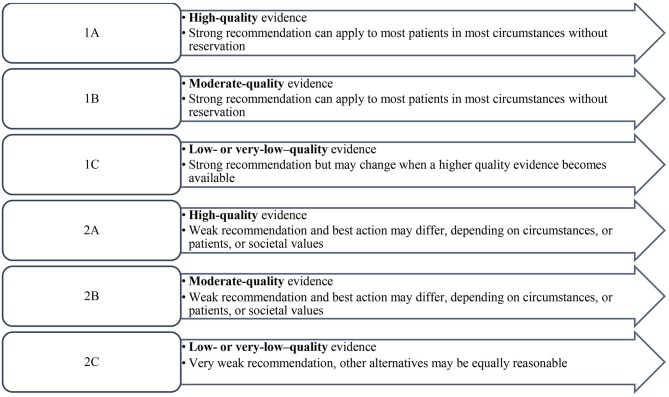
Level of evidence and strength of recommendation.

Descriptive statistics was calculated for each statement to include the mean and median of the responses. The levels of evidence and strength of recommendation were based on the two-level grading system by Guyatt et al. (21) (Figure 1).

## Results

The participating experts critically analyzed existing literature, including randomized clinical trials, systematic reviews, and meta-analyses through a systematic search of MEDLINE (via PubMed), and Cochrane-indexed databases, and guidelines (e.g., NCCN) on CINV management published between 1983 and 2018. A summary of clinical statements with mean score has been provided in Table 1. Consensus was achieved for a total of 12 clinical statements, while 31 statements achieved a near consensus agreement from the experts.

## Discussion

### Chemotherapy-Induced Nausea and Vomiting: A Chink in the Armor of an Oncologist

Chemotherapy-induced emesis exhibits pronounce effects and consequences.

#### Effect and Consequences of Chemotherapy-Induced Nausea and Vomiting

An observational study revealed both acute and delayed CINV to negatively impact a patient's QoL, with delayed variant showing a higher impact on QoL compared to acute CINV (14). In a prospective study, functional status of the patients, as assessed by Functional Living Index-Emesis (FLIE) score [ranging from 0 (not at all affected) to 100 (affected to a great extent)] was considered. A significant increase in the FLIE score for nausea before (day 1) and after chemotherapy (day 5) was observed (6.5 vs. 22.5; *p* < 0.001) (14).

The FLIE scores revealed a significant decline in the functional state of the patient by CINV, particularly in the first 24 h (15). In a retrospective analysis of three studies, results for emesis index from one of the trials showed a significant (*p* < 0.0001), negative effect of CINV on adherence to protocol therapy. Nonadherence to protocol therapy, in turn, affected the survival of the patient [16]. Furthermore, uncontrolled CINV leads to increased resource utilization, thereby increasing the total healthcare cost (11).

### Risk Factors for Chemotherapy-Induced Nausea and Vomiting

The risk factors for CINV can be categorized into: patient-related and chemotherapeutic-agent–related factors.

#### Chemotherapy-Related Risk Factors

The chemotherapeutic agents used alone or in combination trigger different CINV patterns with varying intensity. The NCCN guideline states that for chemotherapies with minimal or low emetic risk, clinicians should avoid overusing antiemetic agents. This will further prevent the patients from adverse effects and reduce the healthcare expenditure (22). Experts recognized that treating oncologists should consider both patient-related and chemotherapy-related risk factors for CINV risk assessment. A good consensus was formed on classifying cisplatin as a highly emetogenic chemotherapy (HEC), irrespective of the dose and regimen and acknowledging Adriamycin-cyclophosphamide (AC) combination as HEC, instead of high-risk moderately emetogenic chemotherapy (MEC), as categorized by the NCCN guideline.

#### Patient-Related Risk Factors

Apart from the chemotherapeutic regimen administered, evidence suggests certain patient-related risk factors to form an integral part of the overall emetic risks for a patient receiving chemotherapy (23–27). Study conducted among chemotherapy-naive patients of phase II and III trials revealed increased nausea in both acute and delayed phases, as the number of risk factors increases. Treatment failure (any emetic episodes or administration of any rescue medication) was significantly higher in patients with three risk factors compared with patients with no risk factors (acute phase: 46.2 vs. 8.9%, *p* < 0.001; delayed phase: 39.3 vs. 54.2%, *p* < 0.001) (27). Furthermore, female gender, nonhabitual alcohol intake and age of <55 years are significant patient-related risk factors for CINV, as they are associated with treatment failure in the acute CINV phase (27). In another observational study conducted in patients undergoing HEC or MEC chemotherapy, female gender was identified as a major prognostic risk factor for CINV [odds ratio (OR): 3.087, 95% confidence interval (CI): 2.219–4.295; *p* < 0.0001]. Older age from both genders was associated with a decrease in acute and delayed CINV (*p* < 0.0001). Furthermore, alcohol intake was found to be associated with decreased risk of delayed CINV (*p* = 0.003), particularly in men (28). High alcohol intake is thought to affect the chemoreceptor trigger zone, thereby having a less pronounced effect by the chemotherapeutic agents (29).

Results from a double-blind, randomized trial showed female gender and age <60 years as significant risk factors (30). A longitudinal observational study echoed similar results of female gender along with other patient-related risk factors to be significant risk factors for CINV. However, the study did not identify young age as a significant risk factor for CINV (31). Strong consensus was formed by the experts on female gender having significantly higher risk of both acute and delayed CINV compared to males. However, a near consensus agreement was built on the increased risk of CINV at young age and decreased CINV risk with alcohol use.

Apart from age and gender, evidence from a univariate analysis in a study revealed history of morning sickness (OR: 2.111, 95% CI: 1.634–2.728; *p* < 0.0001) and motion sickness (OR: 2.796, 95% CI: 2.069–3.778; *p* < 0.0001) in women to be high-risk patient-related factors for acute CINV. Morning sickness was also significantly related to high risk of delayed CINV (*p* < 0.0001) (28). In a prospective study, motion sickness and history of morning sickness experienced in pregnancy were the key prognostic risk factors for CINV (32). In a *post-hoc* analysis, history of morning sickness associated with pregnancy, or morning sickness, contributed as significant patient-related factors in increasing CINV risk (33).

In line with the literature, reasonable consensus came from the experts on the predictors of CINV, such as previous history of motion sickness and morning sickness associated with pregnancy. Psychological factors cannot be ruled out while assessing the risk factors for CINV. Patients' past experiences with CINV can govern and influence response expectancy of nausea for their upcoming chemotherapy (34).

Evidence from a registry trial showed high level anxiety pre-chemotherapy to be a strong predictor of anticipatory CINV in the first cycle of the chemotherapeutic regimen. Patients who experienced CINV in the previous cycle had 3.7 and 3.3 times more chances to develop anticipatory CINV in Cycles 2 and 3, respectively, compared to those who had no prior CINV experience (35). Furthermore, the likelihood of CINV was increased by 6.5 times in Cycle 2, and 14 times in Cycle 3 through the uncontrolled CINV in the previous cycle (35).

A good consensus was obtained on increased risk of CINV with increased anxiety and history of CINV in previous chemotherapeutic cycles. Furthermore, experts acknowledged that CINV risk is high in the first two cycles of chemotherapy, and pain and cancer-related fatigue increase the patients' risk of CINV. Hence, optimum care should be exercised in the first two chemotherapeutic cycles.

Apart from patients' anxiety, role of family as an influencer to CINV episode cannot be ruled out. Finding from a prospective study revealed family support to have a direct impact on the severity of anticipatory CINV. The result from the study suggested that communicating with families might be beneficial in reducing CINV symptoms (34).

A good consensus was achieved for the role of family in CINV risk occurrence, validating the need for patient's family education and counseling. Poor sleep quality and insomnia emerged as other strong predictive risk factors for CINV. Results from an observational study revealed CINV to be significantly associated with poor sleep quality (OR: 2.48, 95% CI: 1.13–5.46; *p* = 0.024) (36). A prospective multicenter, multivariate analysis identified another important independent risk factor for delayed CINV—Eastern Cooperative Oncology Group (ECOG) performance status ≥1 in acute phase (OR: 2.23, 95% CI: 1.04–4.78; *p* = 0.04) (37).

Experts duly acknowledged the increased risk of CINV if the patients received concomitant radiotherapy along with chemotherapy and lack of adequate sleep, a night before the scheduled chemotherapy. A fair consensus was built among the experts, in support for the patients with poor performance status (ECOG) to be an independent risk factor for CINV.

In Asian countries, including India, there is significant usage of alternative medicine (traditional medicine systems) in cancer patients with or without allopathy. However, there is limited evidence concerning their safety and efficacy; a few herbs can interact with the chemotherapeutic agent, leading to several adverse reactions (38). Experts had a near consensus agreement on increased CINV risk in patients on alternative (homeopathic/ayurvedic) medications. Hence, clinicians should also educate and accordingly exercise caution to patients on their use. In absence of robust scientific evidence, no consensus was achieved on the correlation of acute and delayed CINV with history of smoking.

### Management of Chemotherapy-Induced Nausea and Vomiting

Antiemetic regimens are selected based on the drug with the highest emetic risk as well as patient-specific risk factors. The guideline further acknowledges that the risk of nausea/vomiting in patients receiving HEC or MEC lasts for at least 3 days, and 2 days for HEC and MEC settings, after the last dose of chemotherapy. Hence, patients need to be protected throughout the full duration of risk (22). Experts had a good agreement on the recent classification of various intravenous chemotherapeutic agents, according to their emetogenic potential, by the NCCN guideline. A fair consensus surfaced for avoiding prophylaxis of CINV in patients on the minimally emetogenic regimen.

#### Various Antiemetic Agents

##### 5-Hydroxytryptamine (5-HT3) receptor antagonists

As serotonin plays an integral role in the pathophysiology of CINV, 5-HT3 RAs (ondansetron, granisetron, dolasetron, and palonosetron) are invaluable antiemetic agents in the management landscape of CINV (39). The first-generation 5-HT3 RAs are more effective in controlling acute emesis compared to delayed CINV. Based on the scientific evidence, palonosetron has emerged to be a more efficacious and safer 5-HT3 RA agent compared to other agents of the class (39–43). Palonosetron was found to be highly selective, with a strong binding affinity and a long plasma elimination half-life. It has shown its efficacy in preventing CINV in both HEC and MEC settings along with other drugs (40, 41, 44, 45).

A prospective observational study in South Indian patients receiving cancer chemotherapy revealed that as compared to ondansetron, palonosetron is clinically more efficient in controlling CINV. Statistically significant difference in antiemetic response to these two types of prophylaxis was observed, palonosetron being more efficient particularly in delayed phase and overall CINV (*p* = 0.006 for delayed phase, and *p* = 0.008 for overall response). Complete response was observed in 82.1 and 65.1% patients in palonosetron and ondansetron groups, respectively (46). In another prospective, randomized, crossover study involving patients aged between 2 and 18 years, no significant difference was observed in the CR rates across both the treatment groups. Therefore, the findings indicated that ondansetron is noninferior to palonosetron, and can be used as alternative antiemetic drugs (47).

However, it is pertinent to specify the cardiac adverse effects of these agents. QT prolongation is a class adverse effect of these agents. In the light of evidence, special attention is warranted for cancer patients with cardiac disease or elderly cancer patients on polypharmacy (48–50). The NCCN guidelines recommend intravenous palonosetron as the preferred 5-HT3 antagonist (22).

For MEC regimen, the NCCN guideline recommends intravenous palonosetron or subcutaneous granisetron extended-release injection as a preferred 5-HT3 RA, along with dexamethasone. The guideline further recommends a triple-drug regimen, containing NK1 RA or olanzapine, or a four-antiemetic drug regimen, including NK 1RA or olanzapine for HEC setting (22). Additionally, the guidelines duly acknowledge the cardiac effects of the 5-HT3 RAs and suggest routine electrocardiogram (ECG) monitoring during treatment with regimens that include 5-HT3 RAs for patients who may have concomitant risk factors for QT prolongation (22). A fair near consensus agreement was formed for palonosetron as the preferred 5-HT3 RA for HEC setting and ECG monitoring to be essential in patients receiving 5-HT3 RAs. However, experts also opined that the fear of QTc prolongation with antiemetics regimen is spurious.

##### Dexamethasone

Evidence collected over the years shows dexamethasone increasing the efficacy of 5-HT3 RAs in MEC and HEC settings. Efficacy of 5-HT3 RA in terms of complete CINV protection, when combined with dexamethasone for acute CINV, ranged from 68 to 92%; for delayed CINV: 47–73% (42, 43, 51, 52). Though the agent is generally effective, in monotherapy or combination therapy, and is typically administered for multiple days after the start of chemotherapy to prevent delayed CINV, it is associated with insomnia, agitation, rashes, gastrointestinal symptoms, and weight gain (52, 53).

The NCCN guideline acknowledges the side effects of dexamethasone, i.e., insomnia, and hence it recommends specific dosing of dexamethasone for both HEC and MEC regimens. For the triple combination (NK1 RA/palonosetron/dexamethasone) regimen of HEC and MEC settings, the dose of dexamethasone was decreased to 12 mg per oral/intravenous (PO/IV) for day 1. For all the HEC regimens, the guideline recommended dexamethasone 8 mg PO/IV daily on days 2–4 (22). A near consensus emerged for prescribing dexamethasone only on day 1 for acute and delayed CINV in patients on low emetogenic chemotherapy (LEC) and minimal emetogenic regimen. Consistent with the evidence and recommendation for reduction of dexamethasone dose, the experts had a fair consensus on reduction in dose of oral dexamethasone (20–12 mg) during co-administration with an NK1 RA (netupitant).

##### NK1 receptor antagonists

Another important and relatively new class of antiemetics are NK1 RAs (aprepitant, fosaprepitant, netupitant, fosnetupitant, and rolapitant). More and more evidence on efficacy and tolerability of NK1RAs are surfacing from Indian region, highlighting safe and efficacious nature of fosaprepitant, and aprepitant formulations in the Indian population in HEC and MEC settings (54–56). A phase III, randomized, double-blind, placebo-controlled trial was performed in Indian pediatric oncology patients aged 1–12 years on MEC or HEC (ondansetron plus dexamethasone, and fosaprepitant). As compared to the patients in the placebo arm, significantly lower number of patients in the fosaprepitant arm required rescue anti-emetics (20 vs. 4%, *p* = 0.0017) (57). Another single center retrospective cohort study from South India revealed that use of single-dose fosaprepitant in combination with palonosetron, and dexamethasone, effectively prevented CINV (CR: 100%) in both HEC and MEC therapeutic regimens (58).

Furthermore, scientific literature has provided good evidence on the effectiveness and safety of netupitant–palonosetron combination in both HEC and MEC settings (59–62). The NCCN guideline recommends NK1 RA to be added to a 5-HT3/dexamethasone regimen for patients receiving MEC anticancer therapy who have additional risk factors, or previous treatment failure with the two-drug regimen. Patients receiving anticancer therapy, with a higher risk of emesis, are at greater risk of emesis and might require the addition of an NK1 RA (22). Furthermore, for the HEC regimen, any NK1 RA could be used in the four-drug regimen on day 1 (olanzapine/NK1 RA/5-HT3/dexamethasone) (22).

##### Olanzapine

Olanzapine is an atypical antipsychotic, which has antagonizing activity against dopamine (D1–D4 brain receptors), 5-HT2a, 5-HT2c, 5-HT3, histamine (H1 receptors), and muscarinic receptors (63). In a randomized, controlled, Indian trial conducted among 100 chemotherapy-naïve patients on any platinum-based chemotherapy received either, palonosetron and dexamethasone combination or olanzapine (10 mg/day). Results revealed patients in add-on olanzapine group to have significantly better control of delayed compared to dual therapy group (CR: 96 vs. 42%; *p* < 0.0001). Additionally, failure of anti-CINV measure was significantly less in add-on olanzapine group compared to dual (4 vs. 26%) (64). In another randomized, prospective trial; olanzapine as a triple therapy component (with palonosetron and dexamethasone) was found to be as effective as safe as aprepitant for controlling CINV in HEC setting (65). Apart from being effective in breakthrough CINV, olanzapine may serve as a cost-effective alternative to aprepitant in HEC setting and also in patients on HEC regimen who fail on NK1 RA therapy (66, 67). In a prospective, randomized, controlled study conducted in a center in North India, olanzapine group (olanzapine, palonosetron, and dexamethasone) was found to be associated with significantly lowered vomiting and severity of nausea than the control group (palonosetron and dexamethasone). In addition, better control of delayed emesis was observed in the olanzapine-treated patients (CR: 42 vs. 96% in the control and olanzapine-treated groups, respectively, *p* < 0.0001), and overall quality of life was better in this group of patients (64).

In line with the evidence, the NCCN guideline recommended olanzapine-containing three-drug or four-antiemetic drug regimens for both HEC and MEC settings. Furthermore, it was stated that olanzapine could be substituted for dexamethasone in patients who are unable to tolerate dexamethasone (22). However, the only dose-limiting side effects associated with the agent are sedation and drowsiness, which can significantly impact the daily activities of a patient (65). A fair near consensus agreement was achieved for olanzapine as the drug of choice in patients with breakthrough CINV. However, experts felt that sedation associated with olanzapine could be useful in the overall management of CINV, as the already distressed cancer patients can get a good amount of sleep. Hence, based on a patient's condition and nature of job, the antiemetic agent should be individualized.

#### HEC and MEC Regimens (Acute and Delayed Phases)

**Figure 2:**
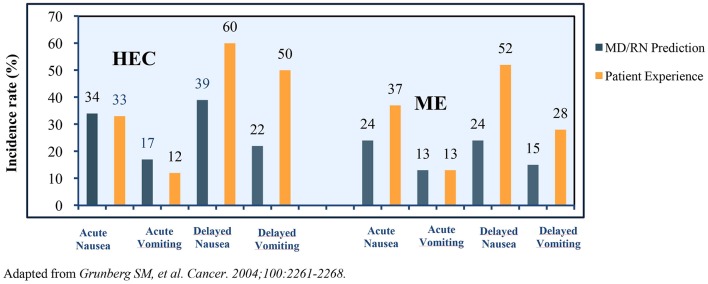
Incidence rates of nausea and vomiting (actual vs. prediction).

Many challenges plague CINV management in patients receiving HEC and MEC regimens. An observational study in patients receiving MEC/HEC for the first time revealed >75% of clinicians and nurses underestimated the incidence of delayed CINV. The representation of the prediction of incidence vs. a patient's experience of CINV variants is presented in Figure 2 (40).

In a retrospective analysis of three clinical trials, various chemotherapy-related toxicities by patients and clinicians were compared. The result from the analysis revealed underreporting of the toxicities (including nausea and vomiting) by the physicians (68).

In a prospective multicenter study, in patients administered HEC/MEC, a significant difference in the control of nausea and CINV was observed in a patient receiving guideline-consistent measures for CINV prevention when compared to patients receiving inconsistent prophylaxis for CINV (CR: 59.9 vs. 50.7%; *p* = 0.008) (9).

In a survey conducted among healthcare providers, significant discrepancies were observed between the recommendations and clinical use of the antiemetic agent in HEC settings with underutilization of NK-1 RAs on day 1 and high use of 5-HT3 RAs on day 2 beyond the chemotherapeutic regimen. There was underutilization of dexamethasone in the MEC setting. The marked uses of phenothiazines (47%) and benzodiazepines (30%) on day 2 and beyond of chemotherapy were found to be inconsistent with the guideline recommendations (69).

Furthermore, the wide range of expected emesis in the MEC regimen (30–90%) makes it challenging to narrow down a specific antiemetic regimen for the whole category (62). In a systematic review conducted by Jordan et al. addition of NK1 RA in a MEC setting exerted a clinically significant benefit in carboplatin-based chemotherapy. The OR obtained for NK1 RA antiemetic regimen for acute and delayed CINV was 1.60 (95% CI: 1.06–2.40; *p* = 0.02) and 2.25 (95% CI: 1.70–2.98; *p* < 0.00001), respectively (62). In Indian scenario, triple therapy with NK1RAs (aprepitant, palonosetron, and dexamethasone) was found to be efficacious and safe with an overall CR rate of 92 and 90.9%, for HEC and MEC regimen, respectively (55). Furthermore, triple therapy with NK1RA was also found to be significantly effective compared to dual therapy (5 HT3 RA+ dexamethasone) in preventing acute and delayed CINV among patients with head and neck cancer (54).

Experts recommended a triple combination therapy (NK1 RA + 5-HT3 RA + dexamethasone) for CINV management in patients receiving AC combination. The experts strongly supported the use of triple combination therapy on day 1 for controlling acute CINV in patients receiving HEC regimen. Compared to olanzapine and dexamethasone combination, the experts had a fair consensus agreement for the use of dual therapy containing NK1 RA and dexamethasone for controlling delayed CINV (days 2–5) in patients receiving HEC.

Experts further acknowledged that triple antiemetic therapy with NK1 RA has the potential to improve outcome in patients on MEC regimen, who have additional risk factors such as female gender, anxiety, motion sickness, etc. Furthermore, for controlling acute CINV in patients receiving a MEC regimen, the consensus was formed on the use of dual therapy (5-HT3 RA + dexamethasone). For patients on MEC, a fair consensus on the use of NK1 RA + dexamethasone or olanzapine + dexamethasone compared to dexamethasone alone was obtained to control delayed CINV (days 2–5).

#### Multiday Chemotherapy

Multiday chemotherapy poses a great management challenge; as the mechanism and pattern of CINV might differ from the single-day chemotherapeutic regimen. Therefore, efficacy of antiemetic agent as observed in single-day chemotherapy may not be extrapolated to the multiday scenario. There is a shortage of data exploring the efficacy and safety of various agents in the specific setting. Patients receiving such regimens are at risk of both acute and delayed CINV (22, 70). As the chemotherapy is extended over several days, it becomes further difficult to specify individual antiemetic agent/regimen for each day of the therapy. However, 5-HT3 RA, dexamethasone, and NK1 RA have greatly improved the management landscape for acute and delayed CINV in multiday chemotherapy (71–73).

There was a good consensus on the use of NK1 RAs to be given only on day 1 for patients who receive multiday chemotherapy. A fair consensus was achieved for 5-HT3 RA daily and palonosetron on days 1, 3, and 5 for patients on multiday chemotherapy. However, no consensus was formed among the experts for the use of long-acting NK1 RA on days 1, 3, and 5 of the multiday chemotherapy. The use of NK1 RA shall further require robust scientific evidence.

## Conclusion and Future Directives

The current evidence indicates that there is still room for improvement concerning CINV management. This document is a sincere effort to address the common unmet needs in CINV-management landscape in the country. The definitive consensus-based clinical statements churned out from the multifaceted approach of the participating experts will guide Indian oncologists to tackle CINV holistically. These statements will also ensure a consistent CINV prevention and management approach in the region.

To further strengthen our discussion, we propose (1) establishing robust resource-stratified guidelines for CINV management, specific to the Indian region, and (2) nation-wide programs to sensitize Indian oncologists toward effective implementation of the drafted guidelines. The CINV guidelines will, in turn, empower the treating oncologists to make informed and individualized decisions on CINV across various healthcare settings. Furthermore, following a consistent preventive and management strategy for the side effect will help in promoting the judicious use of antiemetic agents and, ultimately, help improve the overall QoL in patients undergoing chemotherapy.

## Data Availability Statement

All datasets for this study are included in the article/supplementary material.

## Author Contributions

All authors listed have made a substantial, direct and intellectual contribution to the work, and approved it for publication.

### Conflict of Interest

SB, SP, and HB are employees of Glenmark Pharmaceuticals Ltd. who contributed toward literature search and manuscript writing. The design or procedure of the consensus and the content of the paper are in no way influenced by the grant provider. The authors have no other relevant affiliations or financial involvement with any organization or entity with a financial interest in or financial conflict with the subject matter or materials discussed in the manuscript apart from those disclosed.
